# Formulations of Topical Steroids in Eosinophilic Esophagitis—Current Treatment and Emerging Possibilities

**DOI:** 10.3390/jcm11051454

**Published:** 2022-03-07

**Authors:** Adam Główczewski, Aneta Krogulska

**Affiliations:** Department of Pediatrics, Allergology and Gastroenterology, Ludwik Rydygier Collegium Medicum in Bydgoszcz, Nicolaus Copernicus University, 87-100 Toruń, Poland; klped@cm.umk.pl

**Keywords:** eosinophilic esophagitis, topical steroids, formulation, vehicle

## Abstract

Eosinophilic esophagitis (EoE) is a chronic immune-mediated disorder characterised by eosinophilic inflammation and esophageal dysfunction symptoms. The recommended first-line treatment options are proton pump inhibitors and swallowed topical steroids (STS). However, current recommendations regarding STS are based on relatively few studies employing various doses and formulations. Our aim was to review the STS formulations currently used in the treatment of eosinophilic esophagitis, to demonstrate in a practical way the variety of exiting application methods, and to present emerging options for STS delivery to the esophagus. After the literature review, we established that the three most commonly used STS formulations include mist from an inhaler, viscous suspensions compounded with vehicles for oral use, and a recently introduced proprietary medication in the form of orodispersible tablets. Several drug delivery technologies with potential use in EoE are under investigation. To ensure optimal adherence, the choice of formulation should be based on efficacy, patient preferences and experience of the clinician, as well as current recommendations. Further studies are needed to compare the efficacy and acceptability of existing STS types, and to develop new, well-tolerated and effective drug formulations.

## 1. Introduction

Eosinophilic esophagitis (EoE) is a chronic immune-mediated disease characterised by esophageal tissue eosinophilia and is associated with esophageal dysfunction [1]. Meta-analyses note an overall prevalence of 34.2 cases per 100,000 inhabitants, with a higher rate in adults (42.2/100,000) than in children (34.4/100,000). Although the annual incidence rate is also higher in adults (7.7/100,000 per year) than in children (6.6/100,000 per year), this value is increasing in both groups [2]. Multiple factors are considered to contribute to rising incidence and prevalence, such as environmental factors, microbiome and dietary changes, but also a more widespread use of endoscopy in diagnostics and greater awareness of clinicians, who consider EoE as the reason of esophageal symptoms.

The most distinctive symptoms in adults include dysphagia, food impaction, heartburn, chest pain and acid regurgitation, whereas children most often present with vomiting, abdominal pain, dysphagia and a failure to thrive [3].

A diagnosis of EoE is based on esophageal dysfunction symptoms and the presence of a minimum of 15 eosinophils per high power field (hpf) on esophageal biopsy. According to the guidelines, the biopsies should be multiple (at least 6) and taken from two or more esophageal levels, typically the distal and proximal halves of the esophagus. However, an accurate diagnosis should comprehensively exclude other conditions known to potentially contribute to esophageal eosinophilia, such as non-EoE eosinophilic gastrointestinal disorders, hypereosinophilic syndrome, GERD, achalasia, Crohn’s disease with esophageal involvement, infections, connective tissue disorders, drug hypersensitivity reactions and pill esophagitis [1,4]. Three known risk factors for developing EoE are male gender, Caucasian background and concurrent atopic diseases [2,3,5,6]. 

It is important that therapy should be commenced as soon as possible, as EoE transforms from an inflammatory to a fibrostenotic process, resulting in tissue remodelling and stricture formation. These, in turn, result in increased dysmotility and decreased distensibility, and ultimately, dysphagia and food impaction [7].

Many studies examining the treatment of EoE have focused on the efficacy of swallowed topical steroids (STS) as a first-line option. 

Considering the scarcity of proprietary medicinal products registered for treatment of EoE, the aim of this review is to summarise existing data on the use of STS from a practical point of view, and to provide an overview of existing methods of delivery to the esophagus.

## 2. General Treatment Recommendations for EoE

**Figure 1:**
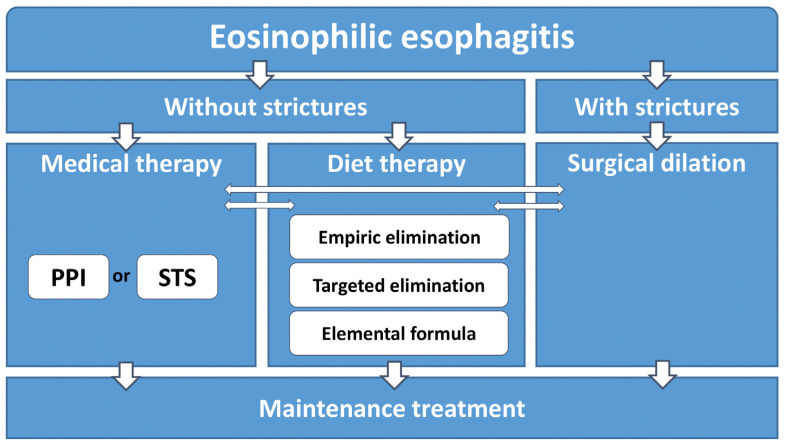
General treatment recommendations in treatment of EoE (PPI—proton pump inhibitors; STS—swallowed topical steroids).

According to current recommendations, first-line treatment should be chosen between medical therapies, such as proton pump inhibitor or STS application, or dietary interventions, such as empiric elimination, elemental formula or targeted elimination [1,8]. In the event that clinically relevant strictures are present at the point of diagnosis, esophageal dilation may also be considered (Figure 1).

After eight weeks of therapy, repeated endoscopy is recommended to assess the mucosal and histological efficacy of the treatment. The aim of treatment is to achieve remission, i.e., resolution of symptoms, mucosal healing and regression of tissue eosinophilia below the threshold of 15 eos/hpf. If remission is achieved, the patient should continue the maintenance treatment.

The real-world data obtained from the EoE Connect database, including data from a large cohort of 589 patients with EoE in Europe, assessed the efficacy of PPI, topical steroids and elimination diets in inducing clinical and histologic remission or response. Of these treatments, the most effective were found to be topical steroids (67.7% patients), followed by empiric elimination diets (52.0%) and PPIs (50.2%) [9].

Despite being effective in inducing remission, it is not recommended to use systemic corticosteroids due to their possible serious adverse systemic effects [1,10]. The only potential use for systemic corticosteroids is as an alternative to surgical dilation for the treatment of esophageal strictures or in a patient with severe symptoms in whom prompt induction of remission is desirable; even then, their use should be restricted to a short course [11]. 

The most commonly chosen first-line treatment option in EoE is PPI administration [9]. However, remission is only achieved in about half of the patients receiving PPIs. This form of treatment is more beneficial in the inflammatory EoE phenotype than the stricturing phenotype, the latter of which is characterised by lower induction and maintenance of remission rates [12]. Even so, PPI therapy is also considered by patients as being more convenient than STS or an elimination diet (ED) [13].

The recommended dietary interventions in EoE comprise an empiric elimination diet, testing-based elimination diet and the use of elemental formulas [1,8]. Elemental diets are based on amino acid-based formulas without any antigenic capacity. They have the highest potential for induction of remission (about 90%); however, they have an unpleasant taste, and their use entails various social limitations caused by the need to avoid all other food [14]. One alternative is the empiric elimination diet. The basic form is the six-food elimination diet (SFED), based on elimination of milk protein, eggs, wheat, soy, peanuts and sea food. Compared to other empiric elimination diets, such as the four-food elimination diet (exclusion of milk protein, eggs, wheat and soy) or the two-food elimination diet (exclusion of milk protein and eggs), the SFED has been proven to be highly effective in inducing remission [14,15,16]. After achieving remission, eliminated allergens should be reintroduced into the diet one by one (step-down approach), and a control biopsy should be performed after each reintroduction to detect the potential food trigger. Alternatively, a step-up empiric elimination approach can be used; in this case, the process begins with two-food elimination, followed by four-food and six-food eliminations, with control endoscopies assessing the effectiveness of each dietary step [17]. Finally, an allergy-testing directed approach (testing-based elimination diet) can be used. In this approach, foods are excluded based on positive results in skin allergy tests. However, this approach has the lowest efficacy (about 45%) for inducing remission in EoE [14]. 

So far, no biologic drugs or anti-allergic agents (such as sodium cromoglicate or antihistamines) have been confirmed to induce or maintain remission in patients with EoE and are not recommended for routine use in this regard [1]. In patients with symptomatic strictures, and in whom anti-inflammatory treatment was not effective, it is recommended to use endoscopic dilation [1,18]. Almost half of the strictures in EoE patients are located in the distal esophagus [19]. Although this method is associated with postprocedural pain, it is both safe and effective, with up to 95% improvement of dysphagia being reported; however, it has no influence on decreasing the ongoing inflammatory process and must be repeated [20,21,22].

As is in many other diseases, the key to achieving remission is adherence to treatment. Any treatment, be it PPI, topical steroids or dietary eliminations, will fail if not applied systematically. A study by Hommel et al. based on parental reports found a high prevalence (reaching 30%) of non-adherence for medication therapy with topical fluticasone in paediatric patients with eosinophilic gastrointestinal disorders (82 out of a total of 96 were diagnosed with EoE). This study also evaluated the non-adherence to dietary treatment. In patients with EoE on elimination diets, the non-adherence prevalence, i.e., at least one exposure to an allergen every two weeks, was 33%. However, the number of missed doses of the drug reported by caregivers was higher in the patients on fluticasone propionate treatment than the number of food exposures in patients on the elimination diet. Regarding age group, medication adherence was poorer in toddlers than in young children [23]. In some cases, treatment non-adherence can result in the development of symptoms of depression [24].

## 3. Mechanism of Activity and Effectiveness of Swallowed Topical Steroids

A major role in the pathogenesis of EoE is played by eosinophils and mast cells, which demonstrate changes in the expression of numerous signaling molecules, including IL-4, IL-5, IL-13, TGF-beta and TSLP, in response to allergens [25]. The later stages of the disease are characterised by fibrosis of the subepithelial layer and the formation of strictures [26]. However, this inflammatory process leading to esophageal remodelling can be slowed by STS treatment [27,28]. Topical steroids are known to have anti-inflammatory properties, which have been attributed to the ability to inhibit pathways induced by IL-13 [29]. Steroid treatment has been shown not only to decrease eosinophil numbers in the esophageal epithelium but also to reduce epithelial cell apoptosis and to decrease esophageal molecular remodelling [30]. STSs have also been found to increase the level of tight junction proteins in the esophageal mucosa of patients with EoE; this correlates with a reduction in the dilation of intercellular spaces, resulting in a potential lower response to food antigens [31]. In addition to eosinophils, STSs also downregulate the response demonstrated by mast cells [32].

The first successful treatment with STSs was reported by Faubion et al. in 1998 [33]. Since that time, their efficacy has been confirmed in further experimental studies and meta-analyses, and STS application has become a recommended first-line option in the induction of histological remission and maintenance treatment; however, studies have yielded varying outcomes due to differences in symptom-scoring methods and their subjective judgement in patients [1,34,35,36,37]. The currently established objective criterion for histologic response to treatment is a threshold of <15 eos/hpf [8].

The two steroids presently recommended for topical use in EoE treatment are budesonide and fluticasone propionate. A retrospective study by Albert et al. compared two groups of patients, comprising both adults and children: one treated with fluticasone propionate (FP) administered from a metered-dose inhaler, and the other with oral viscous budesonide (OVB). The two groups demonstrated similar clinical and histologic responses to the treatments. In contrast, a retrospective study of children by Fable et al. found OVB to be more effective than FP, with respective remission rates of 54% and 35% [38,39].

STSs have been shown to reduce the risk of long-lasting bolus impactions demanding endoscopic removal [40]. STS therapy was found to be the most satisfying approach, with regard to their effectiveness, side effects and convenience, and only a little less convenient than PPI therapy [13]. STS therapy has also proven to be effective in other inflammatory diseases of the esophagus, such as esophageal lichen planus [41].

However, the use of STS is burdened with certain side effects, including oral, pharyngeal and esophageal candidiasis, and an elevated risk of adrenal suppression [42,43,44]. Recently, a single case of cytomegalovirus (CMV) esophagitis was reported in an adult patient with EoE treated with STS [45].

## 4. STS Formulations

Until January 2018, when orodispersible budesonide tablets were approved by the European Medicines Agency, no proprietary steroid-based medicinal products available for topical use were registered for treatment of EoE. Hence, most of the trials were based on pharmaceutical preparations containing steroids obtained from drugs used in the treatment of asthma; these were compounded in pharmacies or by the patients themselves, to be used orally instead of being inhaled. The choice of steroid and the mode of application is usually negotiated between the physician and the patient or guardian. Up-to-date recommendations regarding STS are based on clinical trials conducted with a range of doses and formulas.

The doses of budesonide and fluticasone propionate currently recommended for induction and maintenance treatment in children and adults, are presented in Table 1 (after Lucendo et al. and Savas et al., own modification) [1,34].

To minimise esophageal drug clearance, it is recommended not to eat or drink for at least 30–60 min after administration [1]. A study based on nuclear scintigraphy by Dellon et al. found contact time between the steroid and the esophageal mucosa to play a key role in its effectiveness. A group receiving oral viscous budesonide (OVB) demonstrated significantly greater exposure to the active ingredient (budesonide) than a group receiving nebulized budesonide, and that this exposure correlated with a lower eosinophil count in histopathological assessment [46]. 

In addition, recent investigations suggest that the pharmacokinetics of STS are also influenced by body position and food intake. Gail et al. indicate that STS dosing at bedtime may result in longer mucosal contact with the drug, reflected in a higher maximum serum concentration (Cmax) of fluticasone. Furthermore, the Cmax was higher when following dosing under fed conditions than when fasting [47]. The necessity of regular everyday dosing of STS was supported by Rubinstein et al., who note that every-other-day dosing is not effective in maintaining a histologic response in children and adolescents [48].

### 4.1. Budesonide

Budesonide can be administered in the form of oral viscous budesonide (OVB), a nebulized form, and as orodispersible tablets.

1. Oral viscous budesonide (OVB)

OVB is a slurry mixture obtained from a budesonide suspension designed for nebulisation (available in respules). To improve the consistency and palatability, budesonide is mixed with a vehicle (Table 2).

The standard recipe for OVB is to mix five packets of Splenda^®^, a sucralose-based sweetener, with budesonide respules; this method has been used in many prospective placebo-controlled studies evaluating the efficacy of OVB [30,44,49]. Splenda contains maltodextrin and glucose as filler. 

Sucralose is a non-caloric sweetener with a sweetness potency of about 600 times higher than that of sucrose. Despite many concerns about its potential harmful effect on humans, the evidence suggests that sucralose is safe for use in foods and beverages, both in adults and children [50,51].

In one retrospective study comparing OVB to fluticasone propionate, the included patients were allowed to mix budesonide respules with honey as an alternative to sucralose. However, due to the small sample size, no significant data could be obtained regarding the comparative efficacy of the two vehicles [38].

The OVB slurry mixture can also be prepared using Neocate Nutra as a vehicle. This is a semi-solid amino acid-based hypoallergenic formula designed for infants over six months of age with food allergies. The resulting preparations were found to have similar efficacy to mixtures based on Splenda [52]. 

Another study examined the effectiveness of OVB and FP in patients with EoE. The patients were instructed to mix budesonide respules with five packets of sucralose (Splenda) or with one tablespoon of Duocal^®^, a protein-free high-energy additive for formulas or foods, containing mainly corn syrup solids and refined vegetable oils. Other sweeteners, such as honey, Stevia (a natural sweetener containing stevia leaf extract), Truvia (a sweetener containing stevia leaf extract, erythritol and natural flavours), or a tablespoon of pasteurised maple syrup were also allowed. It was found that the type of delivery vehicle did not have a significant influence on the histologic response to treatment [39]. 

A retrospective cohort study by Reed et al. confirmed that budesonide is an effective treatment when administered as a compounded viscous formulation. The viscous budesonide suspension was prepared by an outpatient compounding pharmacy. The medication consisted of micronized budesonide powder, Methocel E4M Premium (a medium molecular weight hydroxypropyl methylcellulose), a sugar-free sweetener and flavouring agent, at a budesonide concentration of 1 mg/8 mL [53].

A pilot study by Oliva et al. evaluated the efficacy of a pre-prepared formulation of budesonide mainly mixed with the polyalcohol xylitol as a sweetener. The formulation was provided by ITC Farma Srl, and was only for the purpose of the trial, and is not registered as a proprietary medicinal product [54]. In addition, a comparison of various OVB formulations with vehicles based on different sweeteners, such as applesauce, hot cocoa mix, pear sauce, rice cereal and xanthan gum found all to yield similar peak esophageal eosinophil counts [55].

2. Nebulised budesonide

Nebulised budesonide (NB) suspension was found to be effective as an induction therapy and then as a maintenance therapy in two placebo-controlled trials. The suspension was administered by nebulizer. Patients were instructed to administer the suspension into the oropharynx and to continuously swallow the accumulated liquid over 10 min. Dellon et al. found OVB to demonstrate greater effectiveness in inducing remission compared to NB. In addition, patients treated with OVB demonstrated greater esophageal exposure to the therapeutic agent, measured using nuclear scintigraphy, and lower eosinophil counts in esophageal biopsies after treatment [46].

3. Orodispersible tablets

Orodispersible tablets containing budesonide (OBT) are the first proprietary medication to be approved by the European Medicine Agency, designed for EoE treatment. Their efficacy in inducing and maintaining remission has been confirmed in double-blind clinical trials [56,57,58,59]. When placed on the tip of the tongue, OBTs disintegrate on contact with saliva. In addition, as the tablets are effervescent, they stimulate saliva secretion. The dissolved ingredients are then swallowed in small volumes with the saliva over the course of a few minutes until the tablet is fully disintegrated [58].

### 4.2. Fluticasone

Fluticasone can be swallowed from a fluticasone metered-dose inhaler after aerosolization, or derived from a diskus, i.e., a device containing medication as blister packs, or in the form of an oral viscous suspension.

4. Fluticasone metered-dose inhaler (MDI)

The most frequently evaluated method of fluticasone administration in EoE is by metered-dose inhaler (MDI); not only does this method effectively induce remission of the disease, but its simplicity provides greater homogeneity between studies. All patients were instructed to spray the drug (2–4 puffs depending on the prescribed dose) into the oropharynx without the use of a spacer, and then to swallow the aerosolized medication. They were advised to rinse the mouth to avoid any undesirable contact of the drug with the oral mucosa [10,38,39,60,61,62]. The long-term safety of swallowed fluticasone propionate in children, as well as its clinical, histologic and clinic effectiveness in maintenance therapy were demonstrated in an open-label, prospective, single-centre study. The results did not show any growth impediment during the treatment period, with a mean follow-up time of 20.4 months (and the longest, 5.7 years). Three of the 54 studied patients demonstrated esophageal candidiasis; however, this was resolved with anti-fungal therapy [63]. 

Fluticasone propionate has been found to demonstrate 34-times lower bioavailability than budesonide [64].

5. Fluticasone powder

A retrospective analysis by Kia et al. found fluticasone powder administered from a diskus device used as an inhaler in asthmatic patients demonstrated its clinical and histologic efficacy in EoE.

The patients extracted the strip of blister packs from the diskus, peeled back the foil lining and swallowed the powder. The doses varied from 500 to 1000 mcg (2–4 packets, 250 mcg each) [65]. Swallowing the powder appears a more efficient method than swallowing the aerosolized mist from the MDI, as the drug is delivered to the esophagus rather than the nasal passages or airway.

6. Oral viscous fluticasone

Although OVB is the most-evaluated form of delivering budesonide, few studies have examined this approach for fluticasone. Nevertheless, a retrospective cohort study by Ketchem et al. indicates that oral viscous fluticasone propionate has clinical and histologic efficacy in EoE. The medication was dispensed by a specialty compounding pharmacy and was prepared as a viscous suspension consisting of powdered fluticasone propionate and methylcellulose (Methocel gel) at a concentration of 1 mg/8 mL [66].

7. Orally disintegrating tablets

APT-1101 orally disintegrating tablets are the first proprietary medicinal products containing fluticasone propionate designed for EoE treatment. They are currently in a phase 3 clinical trial; however, their clinical and histologic efficacy were previously confirmed in a 1b/3a clinical trial in adults and adolescents by Hirano et al. Patients were instructed to place the tablet on the tongue and to swallow until it dissolved completely. After dissolution, the patients were asked to rinse the mouth with water to remove any remnants of the tablet from the oral cavity [67]. 

### 4.3. Mometasone

The effectiveness of mometasone in treating EoE was evaluated in a retrospective study based on a group comprising 88% children and 12% adults. The patients used a viscous suspension of mometasone (150 mg/mL), compounded by a pharmacist, once daily. The dosage was based on patient height: 750 mg (5 mL) in patients shorter than 110 cm, 1125 mg in patients measuring 110 to 149 cm and 1500 mg in patients 150 cm or taller. The formula was composed of powder forms of mometasone furoate, hydroxypropyl methylcellulose (Methocel 2%), potassium sorbate, citric acid, stevia, sodium benzoate and a flavouring agent. Histologic response was achieved in 76% of patients, 72% of whom had not previously responded to treatment with other swallowed steroids [68]. 

A series of cases by Bergquist et al. found aerosolized mometasone to demonstrate clinical efficacy in EoE, as indicated by an improvement of dysphagia after two months of treatment. The patients received 200 mcg of aerosolized mometasone furoate (4 doses at 50 mcg per spray) orally four times daily (i.e., after each meal and before bedtime) [69]. Mometasone treatment was also found to improve dysphagia in a double-blind placebo-controlled trial [70].

The most significant advantage of mometasone is its low systemic bioavailability, which offers a better safety profile than fluticasone propionate and budesonide [64]. In addition, it has been found to demonstrate efficacy when dosed once daily, which promotes patient compliance.

### 4.4. Ciclesonide

Ciclesonide was found to be effective in two case series reports including eight patients (seven children and one 18-year-old adult). The patients swallowed 160–320 μg of the steroid from a metered-dose inhaler twice daily. Both histological and clinical improvement were noted in patients [71,72]. In addition, similar to mometasone, ciclesonide has a favourable safety profile, with one of the lowest bioavailability ratings of the STSs discussed in this review [64].

### 4.5. Beclomethasone

A pilot randomised placebo-controlled study found beclomethasone to be effective in treating EoE in adults. The patients swallowed two puffs from an inhalation aerosol (160 µg) twice daily for eight weeks. All patients demonstrated a resolution of clinical symptoms and esophageal eosinophilia [73].

An overview of the oral viscous suspensions and aerosolized steroids used in randomised clinical trials and retrospective studies is given in Table 3 and Table 4 [10,30,38,39,46,49,54,55,56,60,61,62,63,66,74,75,76,77].

## 5. Emerging Delivery Methods

A key consideration in therapy with topical corticosteroids is the time that the drug remains in contact with the mucosa, as this duration is directly associated with histological improvement [46]. Therefore, various modifications of compounded OVB have been developed to improve the local effects of steroid delivery, and these have been subjected to randomised placebo-controlled trials [78,79,80].

The following section presents some examples of emerging STS delivery methods.

### 5.1. EsoCap System

The EsoCap system delivers the medication with drinking water. Briefly, while drinking from a special cup with an applicator, the patient swallows a capsule containing a rolled-up polymer film. During the swallowing process, a mucoadhesive film is pulled out from a slit in the capsule and adheres to the esophagus. The gelatin capsule disintegrates in the stomach. The polymer film can be unrolled from the esophagus by a string fixed to the applicator. The functionality of the system has been confirmed by MR imaging [81]. Later studies found films with higher density to be more acceptable in healthy volunteers since they were easier to swallow. In general, the use of the EsoCap device and the swallowability of the capsule were well tolerated [80]. This could be a promising approach to treating EoE by delivering steroids to the esophagus. Moreover, the mucosal contact time of the film can by varied by changing the polymer construction, and the drug can be targeted to certain sections of the esophagus by sectional drug loading of the film [80].

### 5.2. Fluticasone-Eluting String and Fluticasone-Eluting 3D Printed Ring

Prasher et al. report the development of two esophageal drug delivery platforms, which have been studied so far in porcine models. The first, a fluticasone-eluting string, is a strand covered with fluticasone that can be swallowed by a patient, whereupon it releases the drug along the entire length of esophagus, possibly overnight. A high local level was observed in esophageal tissue, and this persisted for one to three days, with minimal systemic absorption. The second is a fluticasone-eluting 3D printed ring that can be placed in the esophagus, where it continually releases the drug. Ex vivo pharmacokinetic studies found a high local level of fluticasone in esophageal tissue with minimal systemic absorption. This preliminary proof-of-concept study suggests that the technology may be translated to patients with EoE in further investigations [82].

## 6. Conclusions

Swallowed topical steroids (STSs) represent a first-line option in the treatment of eosinophilic esophagitis, and their efficacy has been confirmed in a series of prospective and retrospective studies. They exert their activity though a complex mechanism based on their anti-inflammatory and anti-allergic effects on esophageal tissue. A key challenge in the use of STS concerns the mode of delivery to the esophagus, which should ensure prolonged exposure of the esophageal mucosa to the drug.

The aim of this study was not to compare the effectiveness of various STSs or their formulations, as this has already been carried out in a series of meta-analyses [34,35,83,84,85,86,87]. Rather, it summarizes the current level of knowledge on STS formulations and reviews the range of practical application methods, as their correct use plays a key role in adherence.

The basic formulations of STS include mist from an inhaler, oral viscous suspensions compounded with various vehicles, and orodispersible tablets. To ensure maximal treatment compliance, the choice of formulation should be based on current recommendations, and take into account treatment efficacy, patient preferences, experience of the clinician and the local availability of STS preparations. This paper also presents examples of emerging therapeutic options based on state-of-the-art technologies, which will hopefully allow higher treatment efficacy, greater ease of use and acceptance by patients. Nevertheless, there is still a need for further studies comparing the efficacy and acceptability of STS treatments to fill the existing knowledge gap.

## Figures and Tables

**Table 1:** Doses of STS recommended in EoE.

Drug	Phase of Treatment	Children	Adults
Budesonide	Induction	1–2 mg/day	2–4 mg/day
	Maintenance	1 mg/day	2 mg/day
Fluticasone propionate	Induction	880–1760 mcg/day	1760 mcg/day
	Maintenance	440–880 mcg/day	880–1760 mcg/day

**Table 2:** Vehicles used in preparation of OVB.

Preparation Vehicle
sucralose (Splenda^®®^)
amino acid formula (Neocate^®®^ Nutra)
Duocal^®®^
Truvia^®®^
Methocel E4M Premium (hydroxypropyl methylcellulose)
xylitol
stevia
honey

**Table 3:** Overview of oral viscous suspensions of topical steroids used for EoE in randomised controlled trials (RCT) and other studies.

Steroid	Vehicles Used	Preparation	Dose	Dosing	Period ^†^	Response ^‡^	Study Group	Study Type	Ref.
budesonide	sucralose	budesonide resuples (Pulmicort) mixed with sucralose	1–2 mg (approx. 8 mL solution)	QD	3 months	histologic, clinical	children	randomised, placebo-controlled	[49]
budesonide	proprietary medication in RCT	oral viscous suspension—proprietary medication in clinical trial	0.35–2.0 mg (7–10 mL solution)	QD or BID	12 weeks	histologic, clinical	children	randomised, placebo-controlled	[74]
budesonide	proprietary medication in RCT	oral viscous suspension	1–2 mg	BID	2 weeks	histologic, clinical	adults	randomised, placebo-controlled	[56]
budesonide	sucralose	budesonide respules (Pulmicort) mixed with 5 mg of sucralose	1 mg	BID	8 weeks	histologic, clinical	adults	randomised, comparative	[46]
budesonide	xylitol	budesonide suspended in xylitol	1–2 mg (5–10 mL solution)	BID	12 weeks	histologic, clinical	children	prospective, open-label, not blinded	[54]
budesonide	sucralose, applesauce, honey, cocoa mix, pear sauce, rice cereal, xanthan gum	budesonide respules mixed with sucralose or applesauce, or honey, or other (such as hot cocoa mix, pear sauce, rice cereal, xanthan gum)	0.5–1 mg	BID	6 weeks	Histologic ^§^	children	retrospective, cohort	[55]
budesonide	sucralose (Splenda^®^), Neocate^®^ Duocal, Truvia, Stevia, pasteurised maple syrup, honey	budesonide respules mixed with 5 g of sucralose (Splenda^®^) or one tablespoon of Neocate^®^ Duocal, or 2 packets of Truvia, or 2 packets of Stevia, or one tablespoon of pasteurised maple syrup or honey	0.5–1 mg	BID	8–12 weeks	histologic, clinical	children	retrospective, cohort	[39]
fluticasone	Methocel gel	viscous suspension of fluticasone with Methocel gel	1.5–4 mg daily	no data	8 weeks	histologic, clinical	adults	retrospective, cohort	[66]
budesonide	Splenda^®^, honey	budesonide respules mixed with Splenda or honey	0.5–1 mg	BID	8 weeks	histologic, clinical	children and adults	retrospective, comparative	[38]

^†^ the observation period from start of the treatment to control esophageal biopsy, ^‡^ all the studies did differ with histologic scales and symptom score scales, ^§^ clinical response was not assessed in this study BID—twice daily; QD—once daily.

**Table 4:** Overview of aerolised topical steroids used in EoE in randomised controlled trials (RCT) and retrospective studies.

Steroid	Form	Method of Delivery	Dose	Dosing	Period ^†^	Response ^‡^	Study Group	Study Type	Ref.
budesonide	suspension (Pulmicort)	via inhalation system (light compressor and TIA nebulizer)—swallowing continuously the aerolized liquid	0.5 mg	BID	50 weeks	histologic, clinical ^§^	adults and adolescents	randomised, placebo-controlled	[75]
budesonide	suspension (Pulmicort)	via inhalation system—swallowing the mist continuously for 5 min	1 mg	BID	8 weeks	clinical	adults	randomised, comparative	[46]
budesonide	suspension (Pulmicort)	via inhalation system (light compressor and TIA nebulizer)—swallowing continuously the aerolized liquid	2 mg	BID	15 days	histologic, clinical	adolescents, adults	prospective, open-labelled, not blinded	[30]
fluticasone	fluticasone inhaler	swallowing the mist	880 µg	BID	6 weeks	histologic	adults	randomised, placebo-controlled	[60]
fluticasone	no data	no data	880 μg	BID	3 months	histologic	children and adults	randomised, placebo-controlled	[76]
fluticasone	fluticasone inhaler	swallowing the mist	440 μg	BID	3 months	histologic, clinical	children and adults	randomised, comparative	[62]
fluticasone	fluticasone inhaler	swallowing the mist	220–440 μg	4 times dayily	4 weeks	histologic, clinical	children	randomised, comparative	[10]
fluticasone	fluticasone inhaler	swallowing the mist	440 μg	BID	8 weeks	histologic, clinical	adults	randomised, comparative	[77]
fluticasone	fluticasone inhaler	swallowing the mist	440 μg	BID	8 weeks	histologic	adults	randomised, comparative	[61]
fluticasone	fluticasone inhaler	swallowing the mist	176–440 μg	BID	<4 months	histologic, clinical	children	open-label, prospective	[63]
fluticasone	fluticasone inhaler	swallowing the mist	220–440 μg	BID	8 weeks	histologic, clinical	children and adults	retrospective, comparative	[38]
fluticasone	fluticasone inhaler	swallowing the mist	220–440 μg	BID	8–12 weeks	histologic, clinical	children	retrospective, comparative	[39]

^†^ the observation period from start of the treatment to control esophageal biopsy, ^‡^ all the studies did differ with histologic scales and symptom score scales, ^§^ the study assessed the response to the maintenance treatment, the clinical efficacy was not assessed for the whole group (10 patients only) BID twice daily.

## Data Availability

Not applicable.
